# Hepatic Mitochondrial Oxidative Metabolism and Lipogenesis Synergistically Adapt to Mediate Healthy Embryonic-to-Neonatal Transition in Chicken

**DOI:** 10.1038/s41598-019-56715-1

**Published:** 2019-12-27

**Authors:** Chaitra Surugihalli, Tom E. Porter, Angela Chan, Linda S. Farley, Meghan Maguire, Christine Zhang, Nathan Kattapuram, Muhammed S. Muyyarikkandy, Hsiao-Ching Liu, Nishanth E. Sunny

**Affiliations:** 1 Department of Animal and Avian Sciences, University of Maryland, College Park, MD 20742 USA; 2 Department of Animal Science, North Carolina State University, Raleigh, NC 27695 USA

**Keywords:** Endocrine system and metabolic diseases, Diabetes, Obesity, Metabolic syndrome, Physiology, Biochemistry, Metabolomics

## Abstract

During the normal embryonic-to-neonatal development, the chicken liver is subjected to intense lipid burden from high rates of yolk-lipid oxidation and also from the accumulation of the yolk-derived and newly synthesized lipids from carbohydrates. High rates of hepatic lipid oxidation and lipogenesis are also central features of non-alcoholic fatty liver disease (NAFLD) in both rodents and humans, but is associated with impaired insulin signaling, dysfunctional mitochondrial energetics and oxidative stress. However, these adverse effects are not apparent in the liver of embryonic and neonatal chicken, despite lipid burden. Utilizing comprehensive metabolic profiling, we identify that steady induction of hepatic mitochondrial tricarboxylic acid (TCA) cycle and lipogenesis are central features of embryonic-to-neonatal transition. More importantly, the induction of TCA cycle and lipogenesis occurred together with the downregulation of hepatic β-oxidation and ketogenesis in the neonatal chicken. This synergistic remodeling of hepatic metabolic networks blunted inflammatory onset, prevented accumulation of lipotoxic intermediates (ceramides and diacylglycerols) and reduced reactive oxygen species production during embryonic-to-neonatal development. This dynamic remodeling of hepatic mitochondrial oxidative flux and lipogenesis aids in the healthy embryonic-to-neonatal transition in chicken. This natural physiological system could help identify mechanisms regulating mitochondrial function and lipogenesis, with potential implications towards treatment of NAFLD.

## Introduction

The prevalence of NAFLD is approximately 30% in the general population and over 70% in patients with type 2 diabetes mellitus (T2DM)^1,2^. Dysfunctional mitochondrial energetics, sustained lipogenesis, hepatocellular stress and inflammation are all central features of NAFLD and T2DM^3–5^. However, the metabolic mechanisms leading to these hepatic dysfunctions remain elusive. Hepatic mitochondrial oxidative function encompasses central pathways including β-oxidation, TCA cycle, respiratory chain and ketogenesis, which are vital in supporting gluconeogenesis and lipogenesis. As an inherent response to nutrient and hormonal overstimulation, these networks adapt and remodel during the progression of hepatic insulin resistance^6,7^. Thus, a compensatory induction in ‘mitochondrial oxidative function’ is shown to accompany early stages of hepatic insulin resistance^7–9^. However, with progressive severity of NAFLD and T2DM, certain networks (e.g. ketogenesis, mitochondrial respiratory chain and ATP synthesis) are impaired^3,5,10,11^, while others (e.g. TCA cycle, lipogenesis) stay sustained^10,12,13^. Furthermore, chronic induction of oxidative flux through β-oxidation or TCA cycle can aggravate hepatocellular stress and inflammation in NAFLD, potentially due to the inability of the liver to efficiently dispose and/or store lipids arising from the diet and *de novo* lipogenesis^14–17^. Understanding the factors responsible for the optimal relationship between mitochondrial oxidative function, lipogenesis, hepatocellular stress and inflammation is of significant interest towards the management of NAFLD.

During the embryonic-to-neonatal development period in chicken, the liver presents a rapidly adapting and highly plastic metabolic environment, which transitions from fatty acid oxidation in the embryo to lipogenesis in the neonate^18–21^. While the existence of this metabolic switch is known, the role of the hepatic mitochondrial networks in modulating this process is not clear. Furthermore, despite high rates of lipid oxidation during the embryonic stages and high rates of hepatic lipid accumulation in the neonate (from yolk lipids and lipogenesis), a healthy embryonic-to-neonatal transition ensues with no apparent symptoms of metabolic dysfunction, cellular stress or inflammation in the liver. This is unlike rodent models or humans with NAFLD, where high rates of lipid oxidation and hepatic lipid accumulation is concurrent to hepatocellular stress and inflammation^3,12,14^. We hypothesized that the onset of hepatocellular stress and inflammation is prevented during embryonic-to-neonatal development in chicken because of the optimal coupling between mitochondrial oxidative networks and lipogenesis. Metabolic profiling of hepatic mitochondrial oxidative function and lipogenesis illustrate their dynamic remodeling during embryonic-to-neonatal transition in chicken. More importantly, this occurred along with the simultaneous upregulation of antioxidant defense and further more, without the initiation of hepatocellular stress and inflammation.

## Results

### Remodeling of liver physiology with robust induction of hepatic insulin signaling during embryonic-to-neonatal development

**Figure 1:**
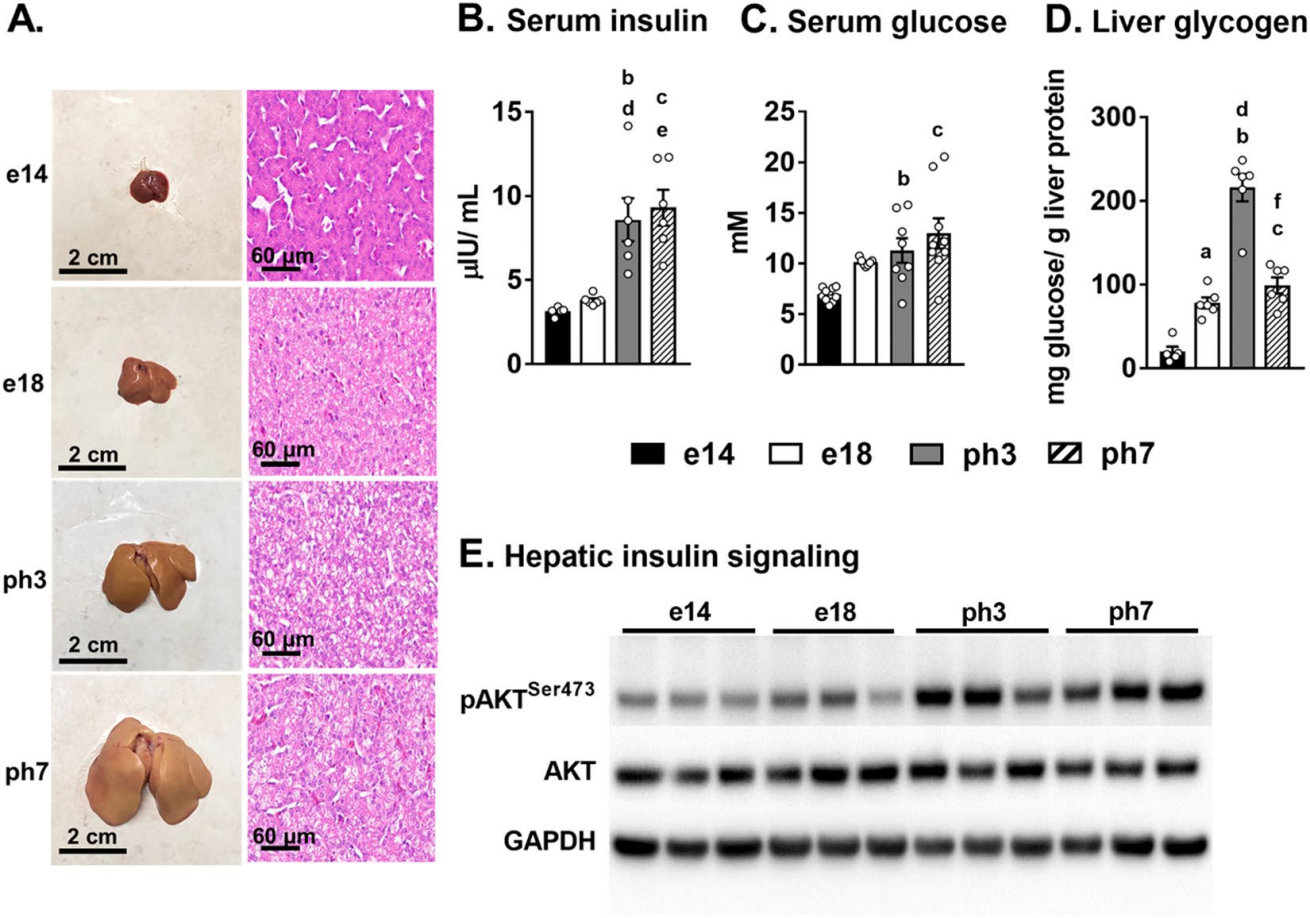
Anabolic adaptations in the liver during embryonic-to-neonatal transition in chicken. (**A**) Changes in liver size and appearance (left) and the corresponding histology (right; n = 3/group) illustrates progressive lipid accumulation. (**B**) Elevated levels of serum insulin in neonatal chicks compared to their embryonic counterparts. (**C**) Progressive increase in serum glucose and (**D**) increase in liver glycogen content from the embryos to the neonates. (**E**) Robust induction of hepatic insulin signaling as evidenced by higher phosphorylation of AKT, during embryonic-to-neonatal transition. Results (n = 6–9/group) were considered significant at p ≤ 0.05 following pairwise mean comparisons, which are represented by the following alphabets. ‘a’- e14 vs. e18; ‘b’ - e14 vs. ph3; ‘c’ - e14 vs. ph7; ‘d’ - e18 vs. ph3; ‘e’ - e18 vs. ph7; ‘f’ - ph3 vs. ph7. AKT, Protein Kinase B; GAPDH, Glyceraldehyde-3-phosphate dehydrogenase.

Supplementary Table S1 details the phenotypic characteristics during embryonic (e14 and 18) and the neonatal stages (ph3 and ph7) in chicken. As the liver size (g ± SEM) increased rapidly from e14 (0.2 ± 0.0) to ph7 (5.7 ± 0.5), the color of the liver grew pale, together with increased accumulation of lipid droplets, evident from the liver histology (Fig. 1A). Furthermore, the transition from embryonic to neonatal stage was characterized by several fold increase in circulating insulin (µIU/mL ± SEM; e14, 3.1 ± 0.1; ph7, 9.3 ± 1.1) (Fig. 1B) and glucose (mM ± SEM; embryonic, 8.3 ± 0.4; neonatal, 12.2 ± 0.9) (Fig. 1C), and also an increase in liver glycogen stores (Fig. 1D), which peaked at ph3 period. These adaptations paralleled an induction of hepatic insulin signaling reflected by the higher phosphorylation of AKT from the embryonic to neonatal stages (Fig. 1E, Supplementary Fig. S1). These results illustrate an ideal anabolic environment for the healthy metabolic transition of the liver from the embryonic to neonatal stage.

### Metabolic switch from free fatty acid oxidation to triglyceride accumulation in the liver is a hallmark of embryonic to neonatal transition in chicken

**Figure 2:**
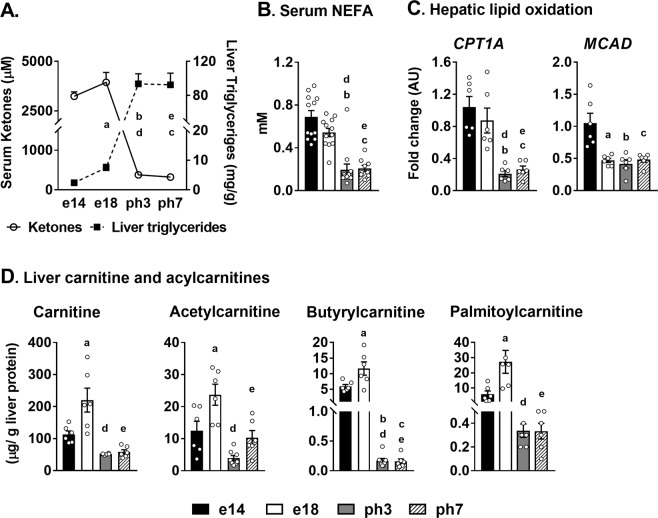
Metabolic switch from free fatty acid utilization/oxidation to triglyceride accumulation in the liver. (**A**) The dramatic reduction in serum ketones from the embryonic period to the neonatal period, concurrent to rapid accumulation of liver triglycerides in the neonatal liver, signifies the metabolic switch from lipid utilization to accretion, (**B**) Serum NEFA levels are significantly lower in the neonatal chicken compared to their embryonic counterparts (**C**) Altered expression of genes associated with hepatic lipid oxidation (*CPT1A* and *MCAD*) substantiates the rapid metabolic switch from hepatic free fatty acid utilization in the embryos to lipid accumulation in the neonates. (**D**) Liver carnitine and acyl carnitines (also see Supplementary Tables S2 and S3 for all the serum and liver acyl carnitine profiles) which fuels high rates of mitochondrial β-oxidation in the embryonic liver were significantly lower in the neonatal liver. Results (n = 6–12/group) were considered significant at p ≤ 0.05 following pairwise mean comparisons, which are represented by the following alphabets. ‘a’- e14 vs. e18; ‘b’ - e14 vs. ph3; ‘c’ - e14 vs. ph7; ‘d’ - e18 vs. ph3; ‘e’ - e18 vs. ph7; ‘f’ - ph3 vs. ph7. NEFA, Non-esterified fatty acids; *CPT1A*, Carnitine palmitoyltransferase 1-alpha; *MCAD*, Medium chain acyl CoA dehydrogenase; AU, arbitrary units.

Serum ketones (mM ± SEM) were high in e14 (3.2 ±0.2) and e18 embryos (3.9±0.5) but significantly dropped in ph3 (0.38 ± 0.04) and ph7 (0.30 ± 0.06) chicks (Fig. 2A). Hepatic triglyceride content (% of liver weight ± SEM) increased significantly from the embryonic period (0.5 ±0.0) to the neonatal period (8.9 ± 1.6) (Fig. 2A, Supplementary Table S1). The drop in serum ketone levels also paralleled a decrease in serum NEFA levels from the embryos to the neonates (Fig. 2B). Further, the lower expression of genes (*CPT1A*, *MCAD*) (Fig. 2C) regulating hepatic lipid oxidation together with lower plasma and hepatic carnitine and acylcarnitine profiles (Supplementary Tables S2 & S3 and Fig. 2D) in the neonatal chicken signified the downregulation of hepatic lipid β-oxidation from embryonic-to-post-hatch. The changes in the AMPK protein expression parallels the extensive lipid oxidation during embryonic stages, especially embryonic day 18, as higher rates of AMPK phosphorylation is reflective of the high energy demands of the rapidly developing embryonic liver (Supplementary Fig. S2).

### The dramatic upregulation of lipogenesis accelerates triglyceride accumulation in the liver

**Figure 3:**
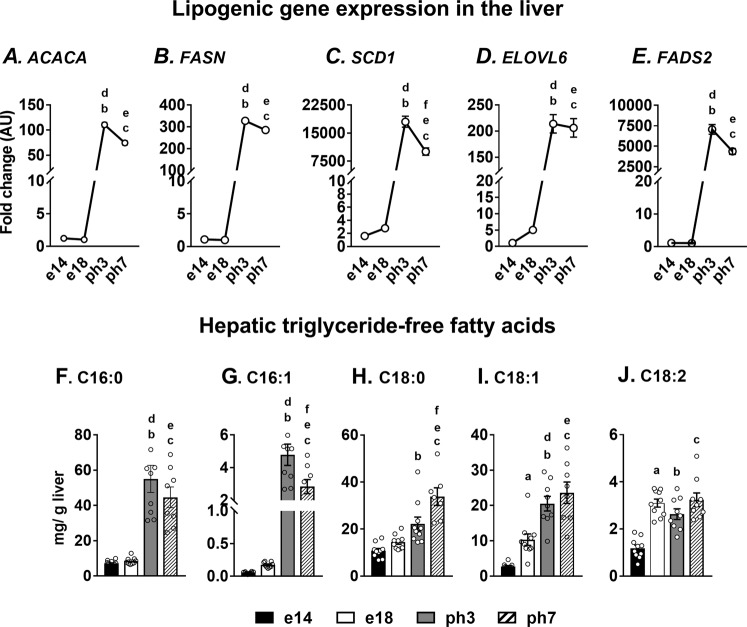
Lipogenesis is a significant contributor to triglyceride accumulation in the neonatal chicken liver. (**A**–**E**) Dramatic upregulation of lipogenic gene expression (*ACACA*, *FASN*, *SCD1*, *ELOVL6*, *FADS2*) provides evidence for the major contribution of hepatic lipogenesis towards lipid accretion. (**F**–**J**) The significant increase in triglyceride-derived free fatty acids (C16:0, C16:1, C18:0, C18:1, C18:2) in neonatal chicks, typically derived from the residual yolk and the starter diet, also contributes to hepatic lipid accumulation. Results (n = 6–10/group) were considered significant at p ≤ 0.05 following pairwise mean comparisons, which are represented by the following alphabets. ‘a’- e14 vs. e18; ‘b’ - e14 vs. ph3; ‘c’ - e14 vs. ph7; ‘d’ - e18 vs. ph3; ‘e’ - e18 vs. ph7; ‘f’ - ph3 vs. ph7. *ACACA*, Acetyl-CoA carboxylase alpha; *FASN*, Fatty acid synthase; *SCD1*, Stearoyl-CoA desaturase 1; *ELOVl6*, Elongation of very long chain fatty acid elongase 6; *FADS2*, Fatty acid desaturase; AU, arbitrary units; C16:0, Palmitate, C16:1, Palmitoleate, C18:0, Stearate, C18:1, Oleate, C18:2, Linoleate.

As there is significant upregulation of lipid accumulation in the liver of the neonate, we profiled the expression of lipogenic genes. *ACACA*, which converts acetyl CoA to malonyl CoA and was upregulated 100 and 75 fold in ph3 and ph7 chickens, respectively (Fig. 3A). Similarly, fatty acid synthase (*FASN*) which facilitates the conversion of malonyl CoA into palmitate was upregulated 300 fold in the neonatal chicken liver (Fig. 3B). Elongation of very long chain fatty acid elongase (*ELOVL6*), which regulates fatty acid elongation, was 200 fold higher in neonates (Fig. 3D). Moreover, fatty acid desaturases, including stearoyl-CoA desaturase 1 (*SCD1*) and the fatty acid desaturase (*FADS2*) were induced several thousand fold in the neonatal liver (Fig. 3C,E). These results illustrate the major role of lipogenesis towards lipid accumulation in the neonatal chicken liver. Furthermore, the triglyceride derived-palmitate, palmitoleate, stearate, oleate and linoleate were all significantly elevated in the liver of neonatal chicken (Fig. 3F–J). Most of these triglyceride derived-fatty acids are derived from the starter diet and the residual yolk lipids, but contributes to the hepatic lipid accumulation in the neonatal liver.

### Hepatic mitochondria in the embryonic and neonatal chicken are primed for substrate oxidation and maintains excellent respiratory control

**Figure 4:**
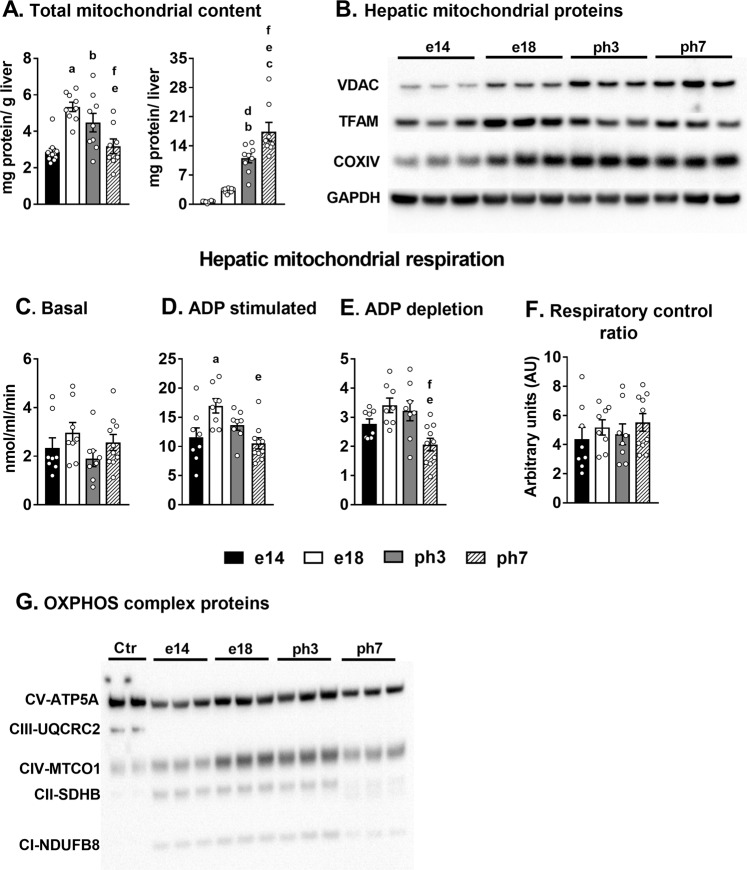
Changes in mitochondrial proteins and Oxphos in the liver during embryonic to neonatal transition. (**A**) Total mitochondrial protein content in the liver expressed on a per gram liver and whole liver basis. (**B**) Changes in expression of hepatic mitochondrial proteins (VDAC, TFAM and COX IV) in the embryos and the neonates. Oxygen consumption by the liver mitochondria determined under (**C**) Basal, (**D**) ADP stimulated and (**E**) ADP depleted states, and the (**F**) Respiratory control ratios in the embryos and the neonates. (**G**) Western blot analysis of changes in the expression of the mitochondrial Oxphos protein complexes (Graphical representation in Supplementary Fig. S3). Results (n = 6–9/group) were considered significant at p ≤ 0.05 following pairwise mean comparisons, which are represented by the following alphabets. ‘a’- e14 vs. e18; ‘b’ - e14 vs. ph3; ‘c’ - e14 vs. ph7; ‘d’ - e18 vs. ph3; ‘e’ - e18 vs. ph7; ‘f’ - ph3 vs. ph7. VDAC, Voltage dependent anion channel; TFAM, Mitochondrial transcription factor A; COX IV, Cytochrome c oxidase subunit 4; Oxphos, Oxidative phosphorylation.

We determined how high rates of lipid oxidation and lipogenesis impacted liver mitochondrial remodeling. Mitochondrial protein content, when expressed per gram liver peaked at e18 and decreased at ph3 and ph7, with the ph7 values similar to those on e14 (Fig. 4A). However, total mitochondrial content, on a whole liver basis continued to increase from e14 to ph7 (Fig. 4A; p < 0.0001). We then determined the expression patterns (per unit of liver protein) of specific mitochondrial proteins in the liver tissue, as an index of mitochondrial content of the liver (Fig. 4B, Supplementary Fig. S3A–C). While the voltage dependent anion channel (VDAC) protein expression showed a steady increase through e14 to ph3, the expression of the mitochondrial transcription factor A (TFAM) and cytochrome c oxidase subunit 4 (Cox IV) plateaued after an initial significant increase from e14 to e18.

To evaluate efficiency and function, mitochondrial oxygen consumption^3,22^ was determined under basal and ADP stimulated conditions. Basal oxygen consumption remained similar between the embryonic and neonatal liver mitochondria (Fig. 4C). However, ADP stimulated respiration, resulted in the highest oxygen consumption rates during e18 (17.9 ± 1.4 nmoles/min), which then tapered to e14 levels (12.7 ± 1.9 nmoles/min) by ph7 (10.5 ± 0.1 nmoles/min; ph3, 14.7 ± 1.3 nmoles/min) (Fig. 4D). Interestingly, the respiratory control ratio (RCR), determined as the ratio of state III (ADP stimulated) to state IV (plateau after ADP stimulation; Fig. 4E) respiration, did not vary and remained high (~4 to 6) through the embryonic-to-neonatal development (Fig. 4F). Further, the expression of the mitochondrial Oxphos complex proteins in the liver were highest during e18 and ph3, and tapered off in the ph7 mitochondria (Fig. 4G; Supplementary Fig. S3D–G).

### Induction of hepatic mitochondrial TCA cycle accompanies high rates of lipogenesis

**Figure 5:**
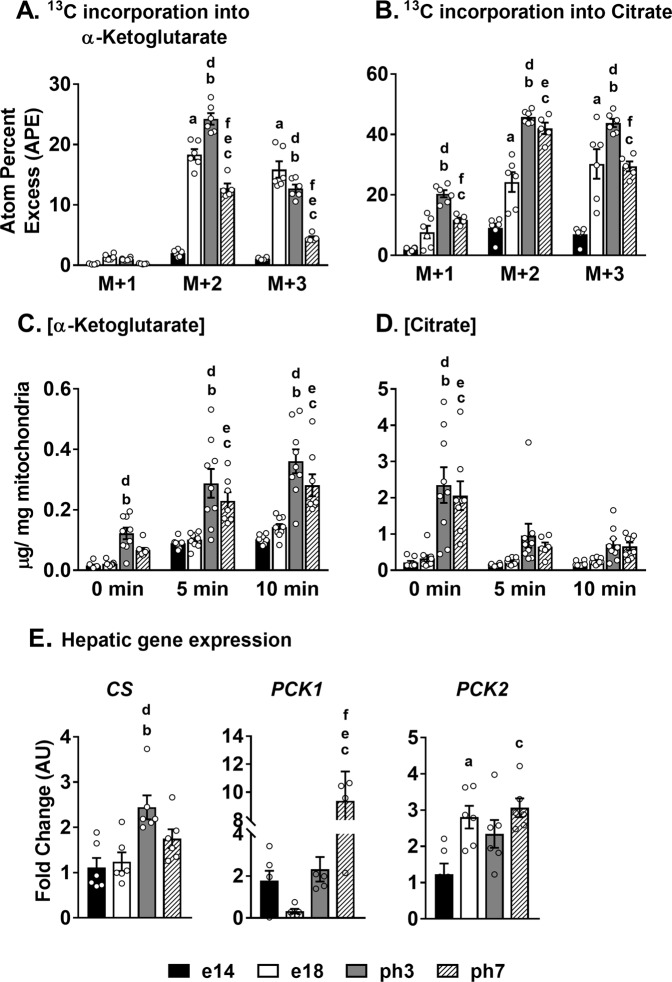
Induction of hepatic mitochondrial TCA cycle metabolism accompany high rates of lipogenesis during embryonic-to-neonatal transition. Increased ^13^C incorporation from [^13^C_3_]pyruvate into (**A**) α-ketoglutarate and (**B**) citrate after 10 min of mitochondrial incubation in e18 and ph3 illustrate elevated mitochondrial TCA cycle activity. Supplementary Tables S4 and S5 provides the ^13^C enrichments in all TCA cycle intermediates following 10 min and 20 min of mitochondrial incubations respectively. Changes in concentrations of (**C**) α-ketoglutarate and (**D**) citrate following incubation of 250 µg of mitochondrial from each group for 0, 5, and 10 minutes (Supplementary Table S6 for the changes in concentrations of succinate, fumarate and malate). (**E**) Changes in the expression of genes involved in the regulation of hepatic TCA cycle metabolism (*CS*, *PCK1* and *PCK2*). Results (n = 6–9/group) were considered significant at p ≤ 0.05 following pairwise mean comparisons, which are represented by the following alphabets. ‘a’- e14 vs. e18; ‘b’ - e14 vs. ph3; ‘c’ - e14 vs. ph7; ‘d’ - e18 vs. ph3; ‘e’ - e18 vs. ph7; ‘f’ - ph3 vs. ph7. *CS*, Citrate synthase; *PCK1*, Phosphoenolpyruvate carboxykinase (cytosolic); *PCK2*, Phosphoenolpyruvate carboxykinase (mitochondrial).

Hepatic TCA cycle plays a critical role in integrating mitochondrial function. To profile TCA cycle metabolism, we incubated isolated mitochondria (250 µg) in respiration buffer containing uniformly labeled [^13^C_3_]pyruvate for 10 and 20 min. The incorporation of ^13^C from pyruvate into TCA cycle intermediates including citrate, α-ketoglutarate, succinate, fumarate and malate was determined using gas chromatography-mass spectrometry (GC-MS). First, there was significant incorporation of ^13^C into all the TCA cycle intermediates for e14, e18, ph3 and ph7 (Fig. 5A,B, and Supplementary Tables S4 & S4). More interestingly, the rate of incorporation of ^13^C was significantly higher (p < 0.05) in metabolites from e18, ph3 and ph7, compared to their e14 counterparts (Fig. 5A,B, and Supplementary Tables S4 & S4). These results illustrates the significant upregulation of hepatic TCA cycle activity between e14 and e18 of development.

We further determined the changes in the ‘pool sizes’ of the TCA cycle intermediates, following 0, 5 or 10 min of incubation of the mitochondria (250 µg) in a respiration buffer. Hepatic mitochondria from ph3 and ph7 had significantly higher (p < 0.05) levels of TCA cycle intermediates compared to their e14 and e18 counterparts (Fig. 5C,D, and Supplementary Table S6). Further, changes in the pool sizes of specific TCA cycle intermediates were also observed with 5 and 10 min of incubation. For example, α-ketoglutarate levels increased steadily with the time of incubation (0 to 10 min) in e14, e18, ph3 and ph7 liver mitochondria (Fig. 5C). Considering the rate-limiting nature of α-ketoglutarate in driving TCA cycle flux^23,24^ this response could point to α-ketoglutarate synthesis from other carbon sources (e.g. amino acids). On the contrary, citrate pool size decreased from 0 to 10 min of mitochondrial incubation, and more interestingly, only in the ph3 and ph7 liver mitochondria (Fig. 5D). Considering the high lipogenic capacity of ph3 and ph7 livers (Fig. 3) and the role of citrate as a precursor for the lipogenic acetyl CoA, changes in citrate pool sizes from 0 to 10 min could reflect the export of citrate out of the mitochondria, as a potential substrate for ATP citrate lyase. A general induction in the expression of genes (e.g. *CS*, *PCK1*, and *PCK2*) involved in the regulation of mitochondrial TCA cycle was also evident from e14 to ph7 (Fig. 5E), substantiating the overall induction of the TCA cycle activity.

### Simultaneous upregulation of hepatic lipogenesis and TCA cycle did not induce inflammation or oxidative stress

**Figure 6:**
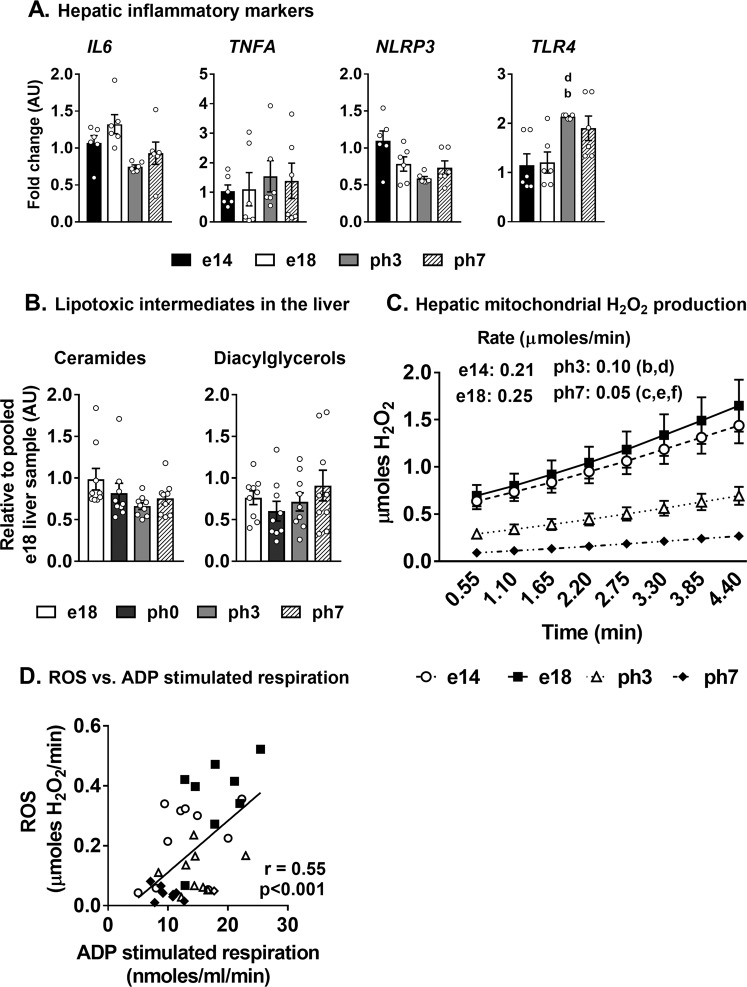
Induction of lipogenesis and mitochondrial TCA cycle, did not induce inflammation or oxidative stress in the liver. (**A**) Expression patterns of inflammatory genes (*IL6*, *TNFA*, *NLRP3* and *TLR4*) remained similar during embryonic-to-neonatal transition. (**B**) Lipotoxic intermediates, Ceramides (left) and Diacylglycerols (right) did not increase with triglyceride accumulation in the liver. (**C**) Reactive oxygen production rates, determined utilizing isolated mitochondria, were significantly lower in neonatal chicks (ph3 and ph7) compared to their embryonic counterparts. (**D**) Correlation between mitochondrial H_2_O_2_ production and ADP stimulated respiration in chicken embryos and neonates. Results (n = 6–9/group) were considered significant at p ≤ 0.05 following pairwise mean comparisons, which are represented by the following alphabets. ‘a’- e14 vs. e18; ‘b’ - e14 vs. ph3; ‘c’ - e14 vs. ph7; ‘d’ - e18 vs. ph3; ‘e’ - e18 vs. ph7; ‘f’ - ph3 vs. ph7; ‘g’ - e18 vs. ph0; ‘h’ - ph0 vs. ph3; ‘i’- ph0 vs. ph7. AU, Arbitrary units; *IL6*, Interleukin 6; TNFA, Tumor necrosis factor A; *NLRP3*, NACHT, LRR and PYD domains-containing protein 3; *TLR4*, Toll like receptor 4.

High TCA cycle activity and sustained lipogenesis during NAFLD and T2DM, co-exist with inflammation and hepatocellular toxicity^13,15,16^. However, during embryonic-to-neonatal transition in chicken, despite high rates of lipogenesis and TCA cycle activity, canonical markers of inflammation (*IL6*, *TNFA*, *NLRP3*) did not display significant changes in their gene expression in the liver (Fig. 6A). However, there was a statistically significant induction of hepatic *TLR4* gene expression in the ph3 group (Fig. 6A). Further, ceramides and diacylglycerols, which are considered lipotoxic intermediates^13,25,26^, remained similar across the groups (Fig. 6B). The rates of hydrogen peroxide formation (µmoles/min ± SEM) by isolated hepatic mitochondria remained similar between e14 (0.21 ± 0.03) and e18 (0.25 ± 0.04) but was significantly reduced in ph3 mitochondria (0.10 ± 0.01) with a further reduction in ph7 (0.05 ± 0.00) (Fig. 6C). A robust correlation between the rates of hydrogen peroxide formation and the ADP stimulated respiration (Fig. 6D; r = 0.55, p < 0.001) illustrates the relationship between ROS generation and Oxphos^27^.

### Antioxidant defense systems were upregulated during embryonic-to-neonatal transition

**Figure 7:**
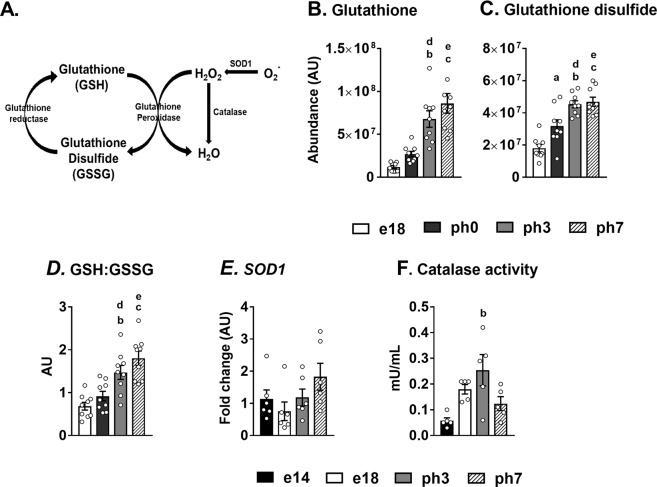
Upregulated antioxidant defense systems during embryonic to neonatal transition. (**A**) General scheme of antioxidant defense mechanisms which helps to reduce the accumulation of reactive oxygen species. (**B,C**) Progressive increase in the levels of glutathione (GSH) and glutathione disulfide (GSSG) which are involved in reducing H_2_O_2_ to water, during embryonic-to-neonatal transition. (**D**) Ratio of GSH: GSSG in embryonic and neonatal chicken (**E**) Expression profile of *SOD1* and (**F**) catalase activity in embryos and neonatal chicks. Results (n = 6–9/group) were considered significant at P ≤ 0.05 following pairwise mean comparisons, which are represented by the following alphabets. ‘a’- e14 vs. e18; ‘b’ - e14 vs. ph3; ‘c’ - e14 vs. ph7; ‘d’ - e18 vs. ph3; ‘e’ - e18 vs. ph7; ‘f’ - ph3 vs. ph7; ‘g’ - e18 vs. ph0; ‘h’ - ph0 vs. ph3; ‘i’- ph0 vs. ph7. GSH, Glutathione; GSSG, Glutathione disulfide; ROS, Reactive oxygen species; *SOD1*, Superoxide dismutase 1, AU, Arbitrary units.

We hypothesized that the antioxidant mechanisms (Fig. 7A) will be upregulated to prevent oxidative stress and inflammation, under a metabolic environment favoring high oxidative activity and lipid accumulation. Indeed, glutathione (GSH), a potent antioxidant and an electron acceptor was progressively increased in the liver from e14 to ph7 (Fig. 7B; p < 0.0001). Similarly, glutathione disulfide (GSSG), an oxidized form of glutathione was also elevated in the neonatal chick liver (Fig. 7C; p < 0.0001). The ratio of GSH to GSSG is commonly used as an indicator of cellular oxidative stress, with a higher ratio indicating lower oxidative stress^28,29^. In fact, the ratio of GSH to GSSG was higher in the neonatal chicks compared to their embryonic counterparts (Fig. 7D), suggesting an induction of antioxidant system and efficient ROS scavenging. Similarly, the trends in superoxide dismutase (*SOD1*) gene expression, even though not statistically significant indicated a pattern of upregulation up to ph7 (Fig. 7E). Catalase activity was the highest during ph3, even though the activity was reduced (p = 0.07) during ph7 (Fig. 7F).

## Discussion

**Figure 8:**
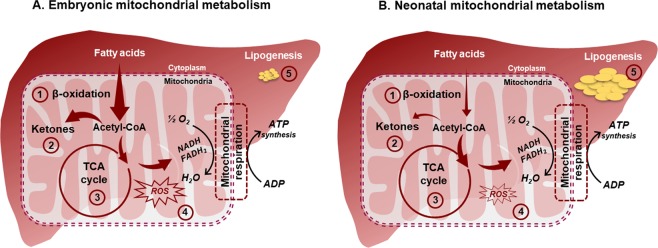
Metabolic shifts which deter hepatic insulin resistance during embryonic-to-neonatal development. Chronic inflammation and hepatic insulin resistance will ensue if the acetyl CoA derived from mitochondrial free fatty acid-β-oxidation cannot be optimally channeled to ketone synthesis or complete oxidation through the TCA cycle. This ‘metabolic bottle neck’ is avoided during embryonic-to-neonatal transition in chicken, through dynamic shifts in mitochondrial β-oxidation, ketogenesis, TCA cycle flux and lipogenesis in the liver. During embryonic development (**A**), high rates of β-oxidation **(1)** generates large amounts of 2-carbon units of acetyl-coA. However, simultaneous upregulation of ketone synthesis **(2)** in the embryonic liver helps to efficiently channel these acetyl-coA units towards other fates. Buffering of acetyl CoA units through ketogenesis during embryonic development occurs concurrent to the steady and progressive induction of hepatic TCA cycle metabolism **(3)**, while rates of lipogenesis remain low **(5)**. After the chicks hatch and starts feeding (**B**), there is a dramatic downregulation of β-oxidation **(1)** and ketogenesis **(2)** in the neonatal liver. The neonatal chicken liver channels the acetyl CoA derived from a carbohydrate rich diet a) through the hepatic TCA cycle for complete oxidation and/or b) exports it from the mitochondria into the cytoplasm as a precursor for lipogenesis **(5)**. In summary, our results suggest that the significant downregulation of β-oxidation after hatch, helps to minimize the metabolic burden from the excess acetyl CoA concurrent to maintaining high rates of lipogenesis and TCA cycle. This prevents high rates of mitochondrial ROS generation in the neonatal liver. **(4)** This dynamic metabolic remodeling during embryonic-to-neonatal transition deter the onset of hepatic insulin resistance.

Dysfunctional mitochondrial networks (e.g. β-oxidation, ketogenesis, TCA cycle flux and Oxphos) and sustained lipogenesis co-exist with hepatocellular stress and inflammation in rodent models and humans with NAFLD and T2DM^15–17^. As several of these networks share biochemical and molecular mediators, it is plausible to hypothesize that synergy between these networks is required to deter hepatic insulin resistance. In embryonic and neonatal chicken, the dynamic but synergistic shifts in mitochondrial β-oxidation, ketogenesis, TCA cycle flux and lipogenesis buffer the metabolic burden from lipids on the liver. This synergy helps avoid hepatocellular stress by efficiently channeling (a) acetyl CoA from free fatty acid oxidation towards ketone synthesis in the embryonic chicken and (b) acetyl CoA from carbohydrate oxidation towards the TCA cycle and lipogenesis in the neonatal chicken (Fig. 8).

The liver of an embryonic chicken has a unique ability to oxidize large amounts of lipids, while the neonatal liver is primed to synthesize, accumulate and transport substantial amounts of lipids^18,21^. The rates of lipid accretion in the neonatal chicken liver (9% of liver weight three days after hatch) is above the threshold (5.5%) considered as NAFLD^30,31^. Our interest is to identify the metabolic mechanisms which allows embryonic and neonatal chicken liver to handle this lipid burden, and in turn undergo healthy development. Further, this is also a relevant question in rodents and humans with NAFLD, as their livers sustain high rates of hepatic lipid oxidation and lipogenesis, but with the side effects of hepatocellular stress and inflammation, in turn aggravating the liver disease^14,15,17^.

An efficient metabolic switch from fatty acid oxidation in the embryonic chicken liver to increased lipogenesis in the neonate, is central for a healthy development^18–21^. Indeed, the embryonic liver mitochondria is primed to oxidize free fatty acids by β-oxidation, as illustrated by the high levels of plasma and hepatic acylcarnitines, higher expression of genes involved in fatty acid oxidation and the high levels of serum ketones (3–4 mM) (Fig. 2, Supplementary Tables S2 & S3). The higher levels of ketones were indicative of the fact that a significant portion of the acetyl CoA derived from β-oxidation were diverted towards the synthesis of ketones. It is also important to note that the synthesis of ketones can serve as a ‘sink’ for acetyl CoA, thus diverting these acetyl CoA carbons from overburdening the hepatic TCA cycle during embryonic development. We believe that this diversion of acetyl CoA towards ketogenesis is aiding the qualitative and quantitative maturation of the mitochondrial TCA cycle and Oxphos in the embryonic liver.

After hatch, along with the depletion of yolk-lipids, the neonatal chicks starts to rely on dietary carbohydrates as the primary metabolic substrates^19^. As a reflection of this, serum ketones fell from 3–4 mM in the embryos to ~300 μM in the neonates, accompanied by a dramatic induction of hepatic lipogenesis (Fig. 3). Circulating ketones could be serving as a substrate depot for initiating hepatic lipogenesis after hatch^32–35^, though the fate of ketones were not tracked in this study. Concurrently, the hepatic TCA cycle in the neonatal chicken is now primed for the complete oxidation of acetyl CoA as indicated by the higher incorporation of ^13^C into mitochondrial TCA cycle intermediates (Fig. 5 and Supplementary Table S4). Furthermore, TCA cycle activity is optimally coupled with mitochondrial respiration as indicated by the steady and high hepatic mitochondrial respiratory control ratios (RCR) (Fig. 4F**)**. More importantly, the metabolic remodeling of lipogenesis and TCA cycle occurred together with the significant downregulation of β-oxidation as signified by the lower levels of plasma and hepatic acylcarnitines, serum ketones and expression patterns of fat oxidation genes in the neonates (Fig. 2, Supplemental Tables S2 & S3). The above results illustrate the dynamic remodeling of hepatic mitochondrial oxidative function and lipogenesis, without initiating inflammation and oxidative stress, as discussed later.

Our results also illustrate the integrative nature of the hepatic TCA cycle, which bring together aspects of mitochondrial oxidative function and lipogenesis. For example, the citrate content of the isolated mitochondria is increasing from e18 to ph3 (Fig. 5D). Furthermore, there is higher incorporation of ^13^C from [^13^C_3_]pyruvate into citrate from e18 to ph3 (Fig. 5B) indicating increased citrate synthesis by the mitochondria. Furthermore, there was a decrease in mitochondrial citrate content from 0 to 10 min, when mitochondria from ph3 and ph7 chicken liver were incubated in a respiration media (Fig. 5D). Taken together, these results point to increased mitochondrial citrate synthesis and higher rates of citrate export from the mitochondria into the cytosol, in order to serve as a lipogenic precursor.

Our results illustrates that the mitochondrial TCA cycle activity and lipogenesis are simultaneously upregulated in the neonatal chicken liver. Interestingly, this occurred without a parallel activation of ROS production or an increase in markers of hepatocellular stress, lipotoxicity or inflammation (Fig. 6). These observations are contrary to those in rodent models and human subjects with NAFLD, where the metabolic milieu favoring high rates of TCA cycle metabolism and lipogenesis coexist with inflammation, hepatocellular stress and lipotoxicity^5,13,15–17^. Increase in ROS occurs when higher amounts of reducing equivalents derived from free fatty acid oxidation (β-oxidation coupled to TCA cycle) drives the transfer of electrons through the mitochondrial respiratory chain^14,27,36^. This process is optimally coupled in embryonic and neonatal chicks, as indicated by the RCR (Fig. 4F). However, a positive correlation between ADP stimulated respiration and ROS production is also evident (Fig. 6D). Coincidently, the highest rates of ADP stimulated respiration and ROS production occurred in the e18 liver when β-oxidation rates were also maximal. These results illustrate that the high rates of reducing equivalents derived from β-oxidation could be significant drivers of ROS production. Thus, the downregulation of β-oxidation immediately post-hatch helped to relieve the oxidative burden from free fatty acids on the hepatic mitochondria, and reduce ROS generation.

During embryonic-to-neonatal development, the reduction in ROS production is associated with an upregulation of antioxidant defense (Fig. 7B,C). The ratio of GSH to its oxidized form, glutathione disulfide (GSSG), a frequently used index of cellular oxidative stress^28,29^ was higher in neonatal chicken (Fig. 7D). This suggests that the induction of antioxidant system and efficient ROS scavenging worked together to deter the adverse impacts of high TCA cycle flux and lipogenesis. Many of the hepatic TCA cycle intermediates have also been shown to have antioxidant activities, through their molecular interactions with antioxidant defense systems^16,37–40^. Indeed, many antioxidant TCA cycle intermediates (e.g. malate, fumarate, citrate) in the liver were progressively higher during embryonic-to-neonatal transition (Supplementary Fig. S4). Taken together, these further highlight the integrative nature of mitochondrial metabolism towards maintaining optimal cellular health and redox state.

In summary, healthy embryonic-to-neonatal transition in the chicken liver is accomplished through the dynamic remodeling of key mitochondrial networks including β-oxidation, ketogenesis and TCA cycle, along with hepatic lipogenesis (Fig. 8). Here, we realize the fact that the embryonic-to-neonatal transition in chicken is an active growth phase, which could have an additional impact on modulating mitochondrial function. Further, there could also be differences in species specific insulin action and glycemic control between the chicken and their mammalian counterparts, which could be contributing to the observed differences. However, the rapid influx of yolk lipids into the liver, coupled together with the fact that liver is the primary lipogenic organ in chicken, makes it an attractive model system to test the impact of lipid overburden. This metabolic milieu is unlike any mammalian system where lipid accumulation and high rates of lipid oxidation are associated with inflammation and hepatocellular stress. Based on our overall results from this natural model, lowering the flux of free fatty acids through β-oxidation could be an effective strategy to reduce mitochondrial ROS and also to avoid the metabolic burden from acetyl CoA on the TCA cycle and Oxphos during NAFLD. Lastly, the embryonic-to-neonatal development period in chicken also presents a unique and natural physiological system to investigate mechanisms regulating hepatic mitochondrial function and lipogenesis.

## Materials and Methods

### Study design

Experiments were conducted in accordance with the Institutional Animal Care and Use Committee protocols approved at the University of Maryland, College Park. Eggs (64 g ± 0.6 standard error of means; SEM) were obtained from Perdue Farms Inc. (Salisbury, MD) from a broiler flock (Ross 708; ~25–30 weeks old), and were incubated at 37 °C, at 45% relative humidity. On the day of hatch (day 21), neonatal chicken were transferred to floor pens maintained at 37 °C and were provided a starter diet (Diet S-G 5065; ASAP Feed and Bedding, Quakertown, PA) ad libitum. Embryonic day 14 and 18 (e14 and e18) and post-hatch days 3 and 7 (ph3 and ph7) were selected for the experiments, with the following rationale. The late term embryonic liver rely predominantly on yolk-lipid oxidation and immediately post-hatch upregulate new lipid synthesis. Thus, the liver during e14 and e18 has high rates of lipid-oxidation and low lipid synthesis, while that of ph3 and ph7 has high lipogenesis and low lipid oxidation. For the studies, embryos were sacrificed by decapitation and, the neonatal chicken were decapitated following isoflurane anesthesia. Blood samples were centrifuged at 1500 × g for 10 min to separate serum. Liver samples were utilized for mitochondrial isolation and a section of the liver was flash-frozen in liquid nitrogen and stored at −80 °C for future analysis.

### Studies on isolated hepatic mitochondria

#### Mitochondrial isolation

Fresh livers were washed with ice cold phosphate buffered saline (PBS; 1X). Tissue (0.5–1 g) was then minced in 2–4 mL MSHE buffer (70 mM sucrose, 210 mM mannitol, 5 mM HEPES, 1 mM EGTA and 0.5% bovine serum albumin (BSA); pH 7.2) and homogenized in a Dounce homogenizer. The homogenate was diluted with 4 mL of MSHE buffer and centrifuged at 800 × g for 10 mins at 4 °C. The supernatant was then passed through a double layered cheese cloth and centrifuged at 8000 × g for 10 mins at 4 °C, to obtain the mitochondrial pellet. This pellet was refined by re-suspending in 3 mL MSHE buffer and centrifuging at 8000 × g for 10 mins at 4 °C, for two times. The final pellet was suspended in 100 µL MSHE buffer without BSA for estimation of mitochondrial protein using Pierce protein assay kit (Thermo Fischer Scientific. Waltham, MA).

#### Isolated hepatic mitochondrial incubations to determine changes in TCA cycle metabolism

Mitochondria (250 µg) was incubated with 1 mM uniformly labeled [^13^C_3_]pyruvate in 1 mL MAS-3 buffer (115 mM KCl, 10 mM KH2PO4, 2 mM MgCl2, 3 mM HEPES, 1 mM EGTA and 0.2% fat free BSA; pH 7.2) containing 5 mM glutamate and 2.5 mM malate. Mitochondrial aliquots from each liver were incubated for 0, 5 and 10 min at 37 °C, following which the mitochondrial pellets were collected and stored at −80 °C for determining the incorporation of ^13^C into the TCA cycle intermediates by gas chromatography- mass spectrometry (GC-MS).

Another set of the mitochondrial samples (250 µg) were incubated as described above without the addition of the stable isotope tracer, to determine the changes in pool sizes of the TCA cycle intermediates. Levels of TCA cycle intermediates in the mitochondria were determined by GC-MS in relation to a known amount of stable isotope labeled internal standards.

#### Hepatic mitochondrial respiration

An oxygraph oxygen electrode (Hansatech Instruments, Norfolk, England) was utilized to measure oxygen consumption by isolated mitochondria (250 µg) suspended in 1-ml of MAS-3 buffer containing 5 mM glutamate and 2.5 mM malate. Basal (state II), ADP stimulated (with 100 μM ADP; state III) and ADP depleted (state IV) respiration rates were determined. Respiratory control ratio (RCR) was calculated as the ratio of state III to state IV respiration^3,41^.

#### ROS generation by isolated hepatic mitochondria

Amplex Red reagent (10-acetyl-3,7-dihydroxyphenoxazine) in combination with HRP (horseradish peroxidase) was used to detect hydrogen peroxide (H_2_O_2_) released by the isolated mitochondria, by recording the real-time oxidation of Amplex red to the florescent resorufin. Mitochondria (15 µg) was incubated with HRP (0.2 U/mL) and amplex red reagent (100 µM) prepared in MAS-3 buffer with glutamate and malate and the changes in fluorescence were detected at 585 nm using a Cytation 5 spectrophotometer (BioTek Instruments, Inc. Winooski, VT).

### Metabolomic analysis

#### GC-MS analysis of mitochondrial and serum metabolites

TCA cycle intermediates were extracted from the mitochondrial pellet or the liver tissue in 750 µL chloroform: methanol (2:1) plus 250 µL water. The aqueous phase was dried and the metabolites were converted to their oximes with 2% methoxamine hydrochloride in pyridine (W/V) by microwaving for 90 sec, and then converted to their respective TBDMS (Tert-butyldimethylsilyl) derivatives^42^.

Serum (25 µL) spiked with [^13^C_4_]β-hydroxybutyrate (500 µM; Cambridge isotopes, MA) were deproteinized with cold acetonitrile, dried and converted to its TBDMS derivative. All the metabolites were separated on a HP-5MS UI column (30 m × 0.25 mm × 0.25 μm; Agilent, CA) and fragmented under electron ionization and detected using single ion monitoring (SIM) on a GC-MS (5973N-Mass Selective Detector, 6890-Series GC, Agilent, CA)^13,42^.

#### GC-MS analysis of triglyceride-free fatty acids

Frozen liver (~20–25 mg) was homogenized with 750 µL chloroform: methanol (2:1) after addition of mixed U^13^C fatty acid standard (Cambridge isotopes, MA). The lipid layer was dried and saponified with 0.5 N methanolic NaOH for 30 mins at 50 °C. Fatty acid methyl esters (FAMEs) were generated with 1 mL of 2% methanolic sulphuric acid and incubation at 50 °C for 2 h, then extracted with 2 mL hexane, dried and re-suspended in 50–100 µL hexane for GC-MS analysis. The FAMEs were separated on a VF 23 ms column (30 m × 0.25 mm × 0.25 μm; Agilent, CA) and fragmented under electron ionization under SIM on a GC-MS (5973N-Mass Selective Detector, 6890-Series GC, Agilent, CA). Concentrations were determined relative to their isotope-labeled internal standard.

#### GC-MS analysis of serum glucose and liver glycogen

Serum (25 μL) and labeled internal standard ([^13^C_6_] glucose; 5.37 mM; Cambridge isotopes, MA) were mixed and deproteinized with 500 μL acetonitrile and dried. The glucose was converted to its Di-O-isopropylidene derivative and separated on a HP-5MS UI column (30 m × 0.25 mm × 0.25 μm; Agilent, CA) under electron ionization (5973N-Mass Selective Detector, 6890-Series GC, Agilent, CA)^42^.

For liver glycogen analysis^42^, frozen liver (~20–25 mg) was deproteinized with 8% sulphosalicylic acid and glycogen was precipitated with cold ethanol. The glycogen pellet was washed with ethanol (2–3 times) to remove residual glucose and the pellet was air dried. The glycogen pellet was incubated with amyloglucosidase in acetate buffer with pH 5.0 (250 μL; 1 unit enzyme/mg glycogen) for 1 h at 55 °C. The released glucose was spiked with ^13^C_6_ glucose internal standard and processed for GC-MS analysis.

#### LC-MS/MS analysis of serum and liver acylcarnitines

Serum (50 µL) and liver (20–25 mg) were homogenized and deproteinized with cold acetonitrile containing a known amount of stable isotope-labeled acylcarnitine internal standard (Cambridge Isotopes, MA), dried and reconstituted in 90:10 methanol-water for liquid chromatography-mass spectrometry (LC-MS/MS). The data was collected using selected reaction monitoring (SRM) mode on a Thermo TSQ Quantum Access triple-quadrupole mass spectrometer with an Accela 1200 LC pump and Heated Electrospray Ionization (HESI) source (positive ionization). Reactions fragmenting to *m/z* 85.3 were monitored following a 5-μl injection on an ACE PFP-C18 column (100 × 2.1 mm, 2 μm particle size) at 40 °C^43^.

#### LC-MS/MS analysis of ceramides (Cer) and diacylglycerols (DAGs) in the liver

Livers from specific pathogen-free (SPF) leghorn chickens (layers) were obtained during the time periods - e18, ph0, ph3 and ph7 days (n = 9)^21^ Following pre-normalization to sample protein concentration (500 μg/ml), samples were Folch extracted and the organic layer was dried and reconstituted for LC-MS/MS analysis. Metabolomics profiling was performed on a Thermo Q-Exactive Oribtrap mass spectrometer with Dionex UHPLC and autosampler. All samples were analyzed in positive and negative heated electrospray ionization with a mass resolution of 35,000 at m/z 200 as separate injections. Separation was achieved on Acquity BEH C18 1.7 μm, 100 × 2.1 mm column for lipid metabolites^13,44^.

#### Liver histology

Livers (~100–150 mg) from embryos and neonatal chicken were fixed in 4% neutral buffered formalin for 18–24 h, washed thrice and stored in 70% ethanol at 4 °C. Hematoxylin and eosin (H&E) staining of liver tissue (n = 3 per group) was performed by Histoserv, Inc., (Germantown, MD) to visualize lipid droplets and/or inflammatory foci.

#### Gene expression analysis

Total RNA was extracted from 20–25 mg of frozen liver using 500 µLTRIZOL reagent (Invitrogen, Carlsbad, CA) and mRNA mini prep kit (Bio-Rad Laboratories Inc., Hercules, CA) following which cDNA was prepared from1 μg mRNA using cDNA synthesis kit (Bio-Rad, Hercules, CA). Quantitative real-time PCR was performed using 25 ng of cDNA, 150 nM of each primer, and 5 μl of SYBR green PCR master mix (Invitrogen, Carlsbad, CA) with GAPDH as housekeeping gene. Samples were run in triplicate on a Bio Rad CFX Real Time system (C1000 Touch Thermal Cycler). For genes with low expression profiles (*IL6* and *TNFA*), gene specific Bio-Rad iselect cDNA synthesis kit (Bio-Rad Laboratories Inc., Hercules, CA) was used to amplify gene expression. The list of primers is provided in the Supplementary Table S7.

#### Western blot analysis

Total liver or mitochondrial protein was determined using pierce BCA protein assay kit (Thermo Fischer Scientific. Waltham, MA). Liver and mitochondrial proteins were separated using Bolt 8% Bis-tris Plus gels (Invitrogen, Carlsbad, CA), transferred to a nitrocellulose membrane and incubated with primary antibodies (Akt, pAkt, COX IV, GAPDH, VDAC (Cell signaling technology Inc., Danvers, MA) and TFAM (Proteintech group, Rosemont, IL)). Total OXPHOS Rodent WB Antibody Cocktail (abcam plc. Cambridge, MA) was used to profile mitochondrial complex proteins involved in oxidative phosphorylation (Oxphos).

#### Biochemical assays

Serum non-esterified fatty acid (NEFA) concentrations were determined using HR Series NEFA-HR2 kit (WAKO diagnostics, CA). Serum insulin was determined by enzyme-linked immunoassay (Cusabio Biotech Co., Ltd., Houston, TX). Liver triglycerides (Serum triglyceride determination kit, Sigma Aldrich, St. Louis, MO) and catalase activity (Catalase Activity Assay kit; abcam plc. Cambridge, MA) were measured according to the manufacturer’s protocol.

### Statistical analysis

All the data reported is presented as mean ± standard error of means (SEM). Results were analyzed using one-way ANOVA, followed by a Tukey’s multiple comparison test. Means were considered significantly different at p ≤ 0.05. Linear regression and correlation analysis were conducted to calculate rates of ROS production (Fig. 6C) and determine the relationship between ROS and ADP stimulated respiration (Fig. 6D). All statistical analysis were conducted and the graphs were plotted utilizing Prism 7 (GraphPad software Inc., San Diego, CA).

## Supplementary information

Supplementary Information.
