# Supplementary material for: Hepatic Mitochondrial Oxidative Metabolism and Lipogenesis Synergistically Adapt to Mediate Healthy Embryonic-to-Neonatal Transition in Chicken
Source: Sci Rep. 2019 Dec 27;9:20167. doi: 10.1038/s41598-019-56715-1 (PMC6934531; doi:10.1038/s41598-019-56715-1)
Supplement: Supplementary file 1 — Supplementary Information. [file 41598_2019_56715_MOESM1_ESM.pdf]

Hepatic Mitochondrial Oxidative Metabolism and Lipogenesis Synergistically Adapt to Mediate Healthy Embryonic-to-Neonatal Transition in Chicken

Chaitra Surugihalli[1], Tom E. Porter[1], Angela Chan[1], Linda S. Farley[1], Meghan Maguire[1], Christine Zhang[1], Nathan Kattapuram[1], Muhammed S. Muyyarikkandy[1], Hsiao-Ching Liu[2], Nishanth E. Sunny[1*]

[1]Department of Animal and Avian Sciences, University of Maryland, College Park, MD 20742, USA

[2]Department of Animal Science, North Carolina State University, Raleigh, NC 27695, USA

*Correspondence and requests for materials should be addressed to N.E.S (e-mail: nsunny@umd.edu), Department of Animal and Avian Sciences, University of Maryland, College Park, MD 20742, USA

	e14	e18	ph3	ph7	p-value
**Embryo weight** (g ± SEM)	13.6 ± 0.32	27.04 ± 0.53			
**Chick weight** (g ± SEM)			60.1 ± 0.81	117.7 ± 5.28	
**Liver weight** (g ± SEM)	0.22 ± 0.00	0.61 ± 0.02	2.50 ± 0.09^{bd}	5.70 ± 0.55^{cef}	<0.0001
**Liver weight** (% body weight)	2.01 ± 0.04	2.29 ± 0.09	4.14 ± 0.13^{bd}	4.78 ± 0.28^{ce}	<0.0001
**% Liver triglycerides**	0.23 ± 0.05	0.74 ± 0.12	9.36 ± 1.79^{bd}	9.24 ± 1.37^{ce}	<0.0001
**Serum insulin** (µIU/mL ± SEM)	3.14 ± 0.11	3.80 ± 0.12	8.58 ± 1.28^{bd}	9.30 ± 1.06^{ce}	<0.0001
**Serum glucose** (mM ± SEM)	6.95 ± 0.21	10.13 ± 0.13	11.29 ± 1.22^{b}	12.98 ± 1.49^{c}	0.0007
**Liver glycogen** (mg glucose/ g liver protein ± SEM)	20.0 ± 6.0	78.1 ± 6.4^{a}	248 ± 35.4^{bd}	99.1 ± 9.6^{cf}	<0.0001

Supplementary Table S1. Metabolic and phenotypic adaptations during embryonic to neonatal transition. n = 6-9/ group; results were considered significant at $p \leq 0.05$ following one way ANOVA and pairwise mean comparisons, represented by the following alphabets. 'a'- e14 vs. e18; 'b' - e14 vs. ph3; 'c' - e14 vs. ph7; 'd' - e18 vs. ph3; 'e' - e18 vs. ph7; 'f' - ph3 vs. ph7.

Serum Acyl carnitines (nmoles/L ± SEM)	e14	e18	ph3	ph7	p-value
Carnitine	101 ± 30.7	262 ± 31.8[a]	144 ± 11.1[d]	145 ± 10.9[e]	0.0001
Acetyl carnitine	60.7 ± 10.2	138 ± 18.9[a]	29.1 ± 2.39[d]	27.9 ± 3.77[e]	<0.0001
Propionyl carnitine	1.16 ± 0.22	4.08 ± 0.51[a]	3.31 ± 0.72[b]	1.90 ± 0.32[e]	0.0030
n-Butyrylcarnitine	0.71 ± 0.14	4.64 ± 0.49[a]	1.07 ± 0.25[d]	0.62 ± 0.22[e]	<0.0001
Butyrylcarnitine	0.90 ± 0.07	4.64 ± 0.49[a]	1.51 ± 0.18[d]	0.93 ± 0.17[e]	<0.0001
Valerylcarnitine	0.21 ± 0.02	0.81 ± 0.08[a]	0.13 ± 0.03[d]	0.06 ± 0.01[e]	<0.0001
Isovalerylcarnitine	0.24 ± 0.03	0.72 ± 0.14[a]	0.55 ± 0.13	0.34 ± 0.06	0.0223
Hexanoylcarnitine	0.08 ± 0.01	0.26 ± 0.06[a]	0.24 ± 0.05	0.18 ± 0.02	0.0264
Octanoylcarnitine	0.03 ± 0.01	0.08 ± 0.02	0.05 ± 0.02	0.04 ± 0.01	0.0827
Myristoylcarnitine	0.63 ± 0.04	0.72 ± 0.05	0.07 ± 0.03[bd]	0.05 ± 0.01[ce]	<0.0001
Palmitoylcarnitine	7.37 ± 0.19	6.96 ± 0.45	0.80 ± 0.28[bd]	1.11 ± 0.20[ce]	<0.0001

Supplementary Table S2. Serum carnitine and acylcarnitine levels during embryonic-to-neonatal transition. n = 6-9/ group; results were considered significant at $p \leq 0.05$ following one way ANOVA and pairwise mean comparisons, represented by the following alphabets. 'a'- e14 vs. e18; 'b' - e14 vs. ph3; 'c' - e14 vs. ph7; 'd' - e18 vs. ph3; 'e' - e18 vs. ph7; 'f' - ph3 vs. ph7.

Liver Acylcarnitines (μg/ g liverprotein ± SEM)	e14	e18	ph3	ph7	p-value
Carnitine	113 ± 10.6	220 ± 37.4[a]	61.1 ± 6.23[d]	58.2 ± 6.89[e]	<0.0001
Acetyl carnitine	12.5 ± 2.98	23.7 ± 3.29[a]	3.94 ± 0.96[d]	10.3 ± 2.24[e]	<0.0001
Propionyl Carnitine	26.9 ± 2.75	28.5 ± 5.19	5.31 ± 0.56[bd]	2.22 ± 0.24[ce]	<0.0001
n-Butyrylcarnitine	5.93 ± 0.62	11.6 ± 2.14[a]	0.17 ± 0.02[bd]	0.14 ± 0.05[ce]	<0.0001
Butyrylcarnitine	5.93 ± 0.62	11.6 ± 2.14[a]	0.17 ± 0.02[bd]	0.16 ± 0.04[ce]	<0.0001
Valerylcarnitine	0.46 ± 0.04	0.70 ± 0.15	0.01 ± 0.00[bd]	0.00 ± 0.00[ce]	<0.0001
Isovalerylcarnitine	1.69 ± 0.18	1.45 ± 0.40	0.06 ± 0.01[bd]	0.04 ± 0.01[ce]	<0.0001
Hexanoylcarnitine	0.08 ± 0.03	0.28 ± 0.06[a]	0.01 ± 0.00[d]	0.02 ± 0.00[e]	<0.0001
Octanoylcarnitine	0.02 ± 0.01	0.15 ± 0.03[a]	0.002 ± 0.001[d]	0.01± 0.00[e]	<0.0001
Myristoylcarnitine	0.17 ± 0.06	0.83 ± 0.24[a]	0.02 ± 0.01[d]	0.01 ± 0.00[e]	0.0003
Palmitoylcarnitine	6.00 ± 2.10	27.2 ± 7.55[a]	0.35 ± 0.08[d]	0.33 ± 0.07[e]	0.0002

Supplementary Table S3. Liver carnitine and acylcarnitine levels during embryonic-to-neonatal transition. n = 6-9/ group; results were considered significant at $p \leq 0.05$ following one way ANOVA and pairwise mean comparisons, represented by the following alphabets. ‘a’- e14 vs. e18; ‘b’ - e14 vs. ph3; ‘c’ - e14 vs. ph7; ‘d’ - e18 vs. ph3; ‘e’ - e18 vs. ph7; ‘f’ - ph3 vs. ph7

Citrate (APE ± SEM)						
	**M+1**	**M+2**	**M+3**	**M+4**	**M+5**	**M+6**
**e14**	1.98 ± 0.29	9.90 ± 1.39	6.99 ± 1.03	3.77 ± 0.58	2.25 ± 0.39	0.91 ± 0.17
**e18**	7.69 ± 2.08	24.24 ± 3.27^{a}	30.26 ± 4.90^{a}	33.72 ± 5.54^{a}	38.59 ± 6.20^{a}	33.78 ± 5.79^{a}
**ph3**	20.37 ± 1.20^{bd}	45.84 ± 0.87^{bd}	43.81 ± 1.36^{bd}	36.72 ± 1.10^{b}	26.15 ± 1.11^{bd}	17.33 ± 1.18^{bd}
**ph7**	11.76 ± 0.69^{cf}	42.01 ± 1.90^{ce}	29.46 ± 1.64^{cf}	17.60 ± 1.21^{cef}	6.76 ± 0.58^{ef}	2.09 ± 0.19^{ef}
**p-value**	<0.0001	<0.0001	<0.0001	<0.0001	<0.0001	<0.0001
**α-Ketoglutarate (APE ± SEM)**						
	**M+1**	**M+2**	**M+3**	**M+4**	**M+5**	
**e14**	0.22 ± 0.03	1.98 ± 0.23	1.09 ± 0.08	0.64 ± 0.05	0.24 ± 0.03	
**e18**	1.34 ± 0.19	18.36 ± 0.89^{a}	15.87 ± 1.35^{a}	19.10 ± 1.72^{a}	23.32 ± 2.42^{a}	
**ph3**	1.01 ± 0.09	24.24 ± 0.96^{bd}	12.70 ± 0.65^{bd}	9.27 ± 0.55^{bd}	6.74 ± 0.69^{bd}	
**ph7**	0.25 ± 0.03	12.78 ± 0.76^{cef}	4.52 ± 0.27^{cef}	2.17 ± 0.14^{ef}	0.50 ± 0.03^{ef}	
**p-value**	<0.0001	<0.0001	<0.0001	<0.0001	<0.0001	
**Succinate (APE ± SEM)**						
	**M+1**	**M+2**	**M+3**	**M+4**		
**e14**	0.30 ± 0.04	2.60 ± 0.20	1.37 ± 0.09	1.17 ± 0.35		
**e18**	-0.09 ± 0.10	6.77 ± 1.03^{a}	3.01 ± 0.58^{a}	9.06 ± 1.82^{a}		
**ph3**	0.56 ± 0.17	13.94 ± 0.94^{bd}	5.47 ± 0.48^{bd}	5.30 ± 0.53^{d}		
**ph7**	0.99 ± 0.86	10.48 ± 0.90^{cf}	3.74 ± 0.96^{ce}	2.82 ± 1.63^{e}		
**p-value**	ns	<0.0001	0.0006	0.0016		

	Fumarate (APE ± SEM)			
	**M+1**	**M+2**	**M+3**	**M+4**
**e14**	1.54 ± 0.15	2.78 ± 0.20	4.77 ± 0.19	1.42 ± 0.05
**e18**	5.45 ± 1.78	14.10 ± 2.72[a]	26.22 ± 4.83[a]	23.93 ± 4.78[a]
**ph3**	0.74 ± 0.17	3.52 ± 0.53[d]	4.85 ± 0.24[d]	3.42 ± 0.16[d]
**ph7**	0.58 ± 0.11	1.83 ± 0.22[e]	1.31 ± 0.05[e]	0.54 ± 0.05[e]
**p-value**	0.0119	<0.0001	<0.0001	<0.0001
	**Malate (APE ± SEM)**			
	**M+1**	**M+2**	**M+3**	**M+4**
**e14**	1.32 ± 0.09	2.09 ± 0.15	3.87 ± 0.16	1.49 ± 0.07
**e18**	4.74 ± 1.39	10.66 ± 2.92[a]	27.91 ± 5.04[a]	24.57 ± 5.29[a]
**ph3**	1.61 ± 0.11	3.92 ± 0.27	6.09 ± 0.32[d]	3.87 ± 0.27[d]
**ph7**	1.11 ± 0.06	2.02 ± 0.07[e]	1.37 ± 0.04[e]	0.54 ± 0.02[e]
**p-value**	0.0068	0.0013	<0.0001	<0.0001

Supplementary Table S4. **Enrichments of TCA cycle intermediates in isolated liver mitochondria from uniformly labeled [$^{13}C_3$]pyruvate, following 10 min of incubation.** n = 6-9/ group; results were considered significant at $p \leq 0.05$ following one way ANOVA and pairwise mean comparisons, represented by the following alphabets. ‘a’- e14 vs. e18; ‘b’ - e14 vs. ph3; ‘c’ - e14 vs. ph7; ‘d’ - e18 vs. ph3; ‘e’ - e18 vs. ph7; ‘f’ - ph3 vs. ph7.

Citrate (APE ± SEM)						
	**M+1**	**M+2**	**M+3**	**M+4**	**M+5**	**M+6**
**e14**	4.93 ± 0.19	20.58 ± 0.96	15.24 ± 0.79	8.99 ± 0.52	5.49 ± 0.42	2.11 ± 0.18
**e18**	7.01 ± 1.57	30.77 ± 2.35^{a}	28.89 ± 3.14^{a}	31.19 ± 3.13^{a}	34.37 ± 3.90^{a}	30.55 ± 4.00^{a}
**ph3**	20.86 ± 1.33^{bd}	47.36 ± 0.90^{bd}	44.58 ± 1.30^{bd}	37.52 ± 0.97^{b}	26.27 ± 0.91^{b}	17.13 ± 1.18^{bd}
**ph7**	9.15 ± 1.63^{f}	37.06 ± 6.14^{cf}	25.84 ± 4.51^{cf}	15.87 ± 2.86^{ef}	6.37 ± 1.19^{ef}	2.17 ± 0.40^{ef}
**p-value**	<0.0001	0.0002	<0.0001	<0.0001	<0.0001	<0.0001

α-Ketoglutarate (APE ± SEM)					
	**M+1**	**M+2**	**M+3**	**M+4**	**M+5**
**e14**	0.49 ± 0.04	7.03 ± 0.16	3.01 ± 0.07	1.75 ± 0.04	0.70 ± 0.02
**e18**	0.91 ± 0.06	22.70 ± 1.21^{a}	13.09 ± 0.66^{a}	16.34 ± 0.90^{a}	17.86 ± 1.53^{a}
**ph3**	0.87 ± 0.08	23.82 ± 0.93^{b}	12.12 ± 0.64^{b}	8.77 ± 0.54^{bd}	6.18 ± 0.62^{bd}
**ph7**	0.35 ± 0.04	12.62 ± 0.30^{cef}	4.48 ± 0.10^{ef}	2.18 ± 0.05^{ef}	0.49 ± 0.01^{ef}
**p-value**	ns	<0.0001	<0.0001	<0.0001	<0.0001

Succinate (APE ± SEM)				
	**M+1**	**M+2**	**M+3**	**M+4**
**e14**	0.81 ± 0.06	6.77 ± 0.42	2.75 ± 0.24	2.18 ± 0.41
**e18**	-0.04 ± 0.18	9.93 ± 1.05^{a}	4.47 ± 0.52	9.94 ± 1.56a
**ph3**	0.60 ± 0.17	15.11 ± 1.23^{bd}	5.83 ± 0.58^{b}	5.56 ± 0.60^{bd}
**ph7**	0.06 ± 0.07	9.45 ± 0.32 ^{cf}	2.73 ± 0.13^{f}	1.47 ± 0.36^{ef}
**p-value**	ns	<0.0001	<0.0001	<0.0001

Fumarate (APE ± SEM)				
	**M+1**	**M+2**	**M+3**	**M+4**
**e14**	2.04 ± 0.18	3.19 ± 0.25	4.97 ± 0.21	1.60 ± 0.07
**e18**	0.82 ± 0.28	6.33 ± 0.93[a]	15.26 ± 2.10[a]	11.12 ± 1.76[a]
**ph3**	0.72 ± 0.11	4.37 ± 0.70	4.67 ± 0.20[d]	3.24 ± 0.26[d]
**ph7**	0.64 ± 0.07	1.91 ± 0.12[e]	1.30 ± 0.06[cef]	0.49 ± 0.04[e]
**p-value**	<0.0001	0.0003	<0.0001	<0.0001
**Malate (APE ± SEM)**				
	**M+1**	**M+2**	**M+3**	**M+4**
**e14**	1.75 ± 0.11	2.75 ± 0.18	4.62 ± 0.19	1.88 ± 0.09
**e18**	1.42 ± 0.18	4.25 ± 0.59	18.56 ± 3.02[a]	12.20 ± 2.50[a]
**ph3**	1.49 ± 0.12	3.90 ± 0.30	5.95 ± 0.27[d]	3.75 ± 0.29[d]
**ph7**	1.16 ± 0.04	2.27 ± 0.08	1.50 ± 0.06[ef]	0.61 ± 0.03[e]
**p-value**	0.0257	0.0018	<0.0001	<0.0001

Supplementary Table S5. Enrichments of TCA cycle intermediates in isolated liver mitochondria from uniformly labeled [$^{13}C_3$]pyruvate, following 20 min of incubation. n = 6-9/ group; results were considered significant at p ≤ 0.05 following one way ANOVA and pairwise mean comparisons, represented by the following alphabets. 'a'- e14 vs. e18; 'b' - e14 vs. ph3; 'c' - e14 vs. ph7; 'd' - e18 vs. ph3; 'e' - e18 vs. ph7; 'f' - ph3 vs. ph

Lactate (µg/ mg mitochondria ± SEM)				
	**0 min**	**5 min**	**10 min**	**p-value**
**e14**	0.41 ± 0.08	0.56 ± 0.01	0.43 ± 0.12	ns
**e18**	0.58 ± 0.17	0.44 ± 0.06	0.51 ± 0.09	ns
**ph3**	0.76 ± 0.24	1.01 ± 0.35^{d}	0.65 ± 0.20	ns
**ph7**	0.79 ± 0.20	0.47 ± 0.17^{f}	0.67 ± 0.18	ns
**p-value**	ns	ns	ns	
**Pyruvate (µg/ mg mitochondria ± SEM)**				
	**0 min**	**5 min**	**10 min**	**p-value**
**e14**	0.14 ± 0.01	0.06 ± 0.01	0.06 ± 0.01	<0.0001
**e18**	0.23 ± 0.03	0.08 ± 0.01	0.09 ± 0.01	<0.0001
**ph3**	0.56 ± 0.07^{bd}	0.30 ± 0.04^{bd}	0.25 ± 0.03^{bd}	0.0006
**ph7**	0.35 ± 0.06^{cf}	0.20 ± 0.04^{c}	0.17 ± 0.03	ns
**p-value**	<0.0001	<0.0001	<0.0001	
**Citrate (µg/ mg mitochondria ± SEM)**				
	**0 min**	**5 min**	**10 min**	**p-value**
**e14**	0.21 ± 0.05	0.15 ± 0.01	0.18 ± 0.02	ns
**e18**	0.33 ± 0.09	0.24 ± 0.03	0.25 ± 0.03	ns
**ph3**	2.35 ± 0.49^{bd}	0.95 ± 0.33	0.72 ± 0.15	0.0066
**ph7**	2.06 ± 0.39^{ce}	1.52 ± 0.77	0.66 ± 0.11	ns
**p-value**	<0.0001	ns	ns	
**α-Ketoglutarate (µg/ mg mitochondria ± SEM)**				
	**0 min**	**5 min**	**10 min**	**p-value**
**e14**	0.02 ± 0.00	0.09 ± 0.01	0.10 ± 0.00	<0.0001
**e18**	0.02 ± 0.00	0.10 ± 0.01	0.14 ± 0.01	<0.0001
**ph3**	0.12 ± 0.02^{bd}	0.29 ± 0.05^{bd}	0.36 ± 0.04^{bd}	0.0005
**ph7**	0.07 ± 0.01	0.23 ± 0.03^{ce}	0.28 ± 0.04^{ce}	<0.0001
**p-value**	<0.0001	<0.0001	<0.0001	
**Succinate (µg/ mg mitochondria ± SEM)**				
	**0 min**	**5 min**	**10 min**	**p-value**

**e14**	0.38 ± 0.08	0.84 ± 0.35	0.42 ± 0.12	ns
**e18**	0.35 ± 0.06	0.50 ± 0.09	0.54 ± 0.09	ns
**ph3**	0.55 ± 0.06	0.59 ± 0.15	0.48 ± 0.07	ns
**ph7**	0.50± 0.07	0.46 ± 0.08	0.38 ± 0.08	ns
**p-value**	ns	ns	ns	
**Fumarate (µg/ mg mitochondria ± SEM)**				
	**0 min**	**5 min**	**10 min**	**p-value**
**e14**	0.32 ± 0.11	1.97 ± 0.16	2.11 ± 0.12	<0.0001
**e18**	0.69 ± 0.12	3.15 ± 0.22[a]	3.13 ± 0.22[a]	<0.0001
**ph3**	2.25 ± 0.22[bd]	3.23 ± 0.31[b]	3.31 ± 0.29[b]	0.0201
**ph7**	1.22 ± 0.17[cf]	2.38 ± 0.34[f]	2.59 ± 0.32	0.0048
**p-value**	<0.0001	0.0041	0.0081	
**Malate (µg/ mg mitochondria ± SEM)**				
	**0 min**	**5 min**	**10 min**	**p-value**
**e14**	0.17 ± 0.08	1.41 ± 0.14	1.61 ± 0.11	<0.0001
**e18**	0.36 ± 0.07	2.46 ± 0.35[a]	2.63 ± 0.19[a]	<0.0001
**ph3**	1.39 ± 0.14[bd]	2.12 ± 0.24[b]	1.99 ± 0.24	0.0541
**ph7**	0.73 ± 0.09	1.74 ± 0.25	1.64 ± 0.17[e]	0.0009
**p-value**	<0.0001	0.0458	0.0018	

Supplementary Table S6. **Concentrations of TCA cycle intermediates in the isolated mitochondria following 0, 5 and 10 min of incubation.** n = 6-9/ group; results were considered significant at $p \leq 0.05$ following one way ANOVA and pairwise mean comparisons, represented by the following alphabets. ‘a’- e14 vs. e18; ‘b’ - e14 vs. ph3; ‘c’ - e14 vs. ph7; ‘d’ - e18 vs. ph3; ‘e’ - e18 vs. ph7; ‘f’ - ph3 vs. ph

Gene	Forward primer	Reverse primer	Accession No.
ACACA	TGGCAGCCATGTTCAGAGAG	GGAAATTCCCTCTTCTGTGCCA	NM_205505.1
CS	AGGGATTTCATCTGGAACACACT	CACCGTGTAGTACTTCATCTCCCT	XM_015300289.2
CPT1A	TGCTCACTACCGAGACATGG	GGTACATGACCGGACGGTTT	XM_015286798.2
ELOVL6	CCGGGCCAATGAACATGT	TCATGCTCGTTGAACTGCTTCT	NM_001031539.1
FADS2	TCTTAATGGGGAGGGAACAGGT	ACCAGCTTCTTGCATTTTCACA	NM_001160428.2
FASN	GGCTACACACTAGTTGGCACT	CACTGTGTTCCCATGCCTGA	NM_205155.3
GAPDH	GATTTAATGAGCCATTCGCAGTT	CCCAGCGTGCATGTCTAAGTAC	NM. 205155.3
IL6	GCAGGACGAGATGTGCAAGA	GTCCTCCTCCGTCACCTT	NM_204628.1
MCAD	CACGCAGTAGGCACACATGAT	CGGCAGCAGCGCAAAT	
NLRP3	GGTTTACCAGGGGAA ATGAGG	TTGTGCTTCCAGATGCCGT	NM_001348947.1
PCK1	GCAGGGGTTATGATGAGAAGT	ACGGATCACAGTTTTGAAGAC	NM_205471.1
PCK2	CCTTCGCCATGAGCCCCTTTTTC	CAGCTCCGCCATGACATCCCT	NM_205470.1
SCD1	CAATGCCACCTGGCTAGTGA	CGGCCGATTGCCAAAC	NM_204890.1
SOD1	GACCAAAAGATGCAGATAGG	TCCAGCATTTCCAGTTAGTT	NM_205064
TLR4	AGTCTGAAATTGCTGAGCTCAAAT	GCGACGTTAAGCCATGGAAG	NM_001030693.1
TNFA	CCATATGACCACGCTCTTTCCG	AGCAGCAGCAGCAGCAGAGC	MF000729.1

Supplementary Table S7. List of primers used for RT-qPCR.

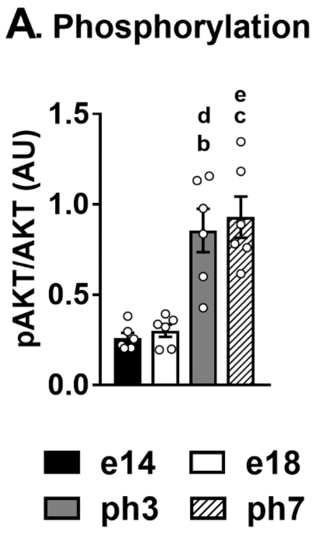

Supplementary Fig S1. **Graphical representation of AKT phosphorylation in the liver.** Densitometry of western blots for phosphorylated AKT (Ser473) relative to total AKT (from Fig 1E). n = 6/ group; Means were considered significant at $P \leq 0.05$ following pairwise mean comparisons, represented by the following alphabets, 'a'- e14 vs. e18; 'b' - e14 vs. ph3; 'c' - e14 vs. ph7; 'd' - e18 vs. ph3; 'e' - e18 vs. ph7; 'f' - ph3 vs. ph7.

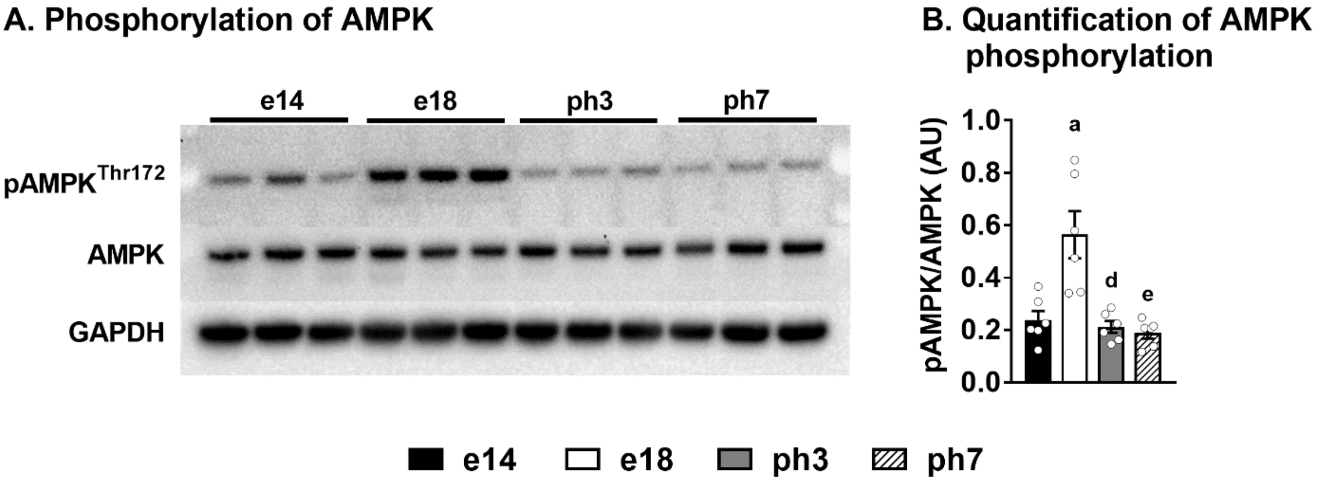

Supplementary Fig S2. AMPK phosphorylation in the liver. (**A**) Western blot and (**B**) Densitometry of the blots for phosphorylated AMPK (Thr172) relative to total AMPK. n = 6/ group; Means were considered significant at P ≤ 0.05 following pairwise mean comparison, represented by the following alphabets, ‘a’- e14 vs. e18; ‘b’ - e14 vs. ph3; ‘c’ - e14 vs. ph7; ‘d’ - e18 vs. ph3; ‘e’ - e18 vs. ph7; ‘f’ - ph3 vs. ph7.

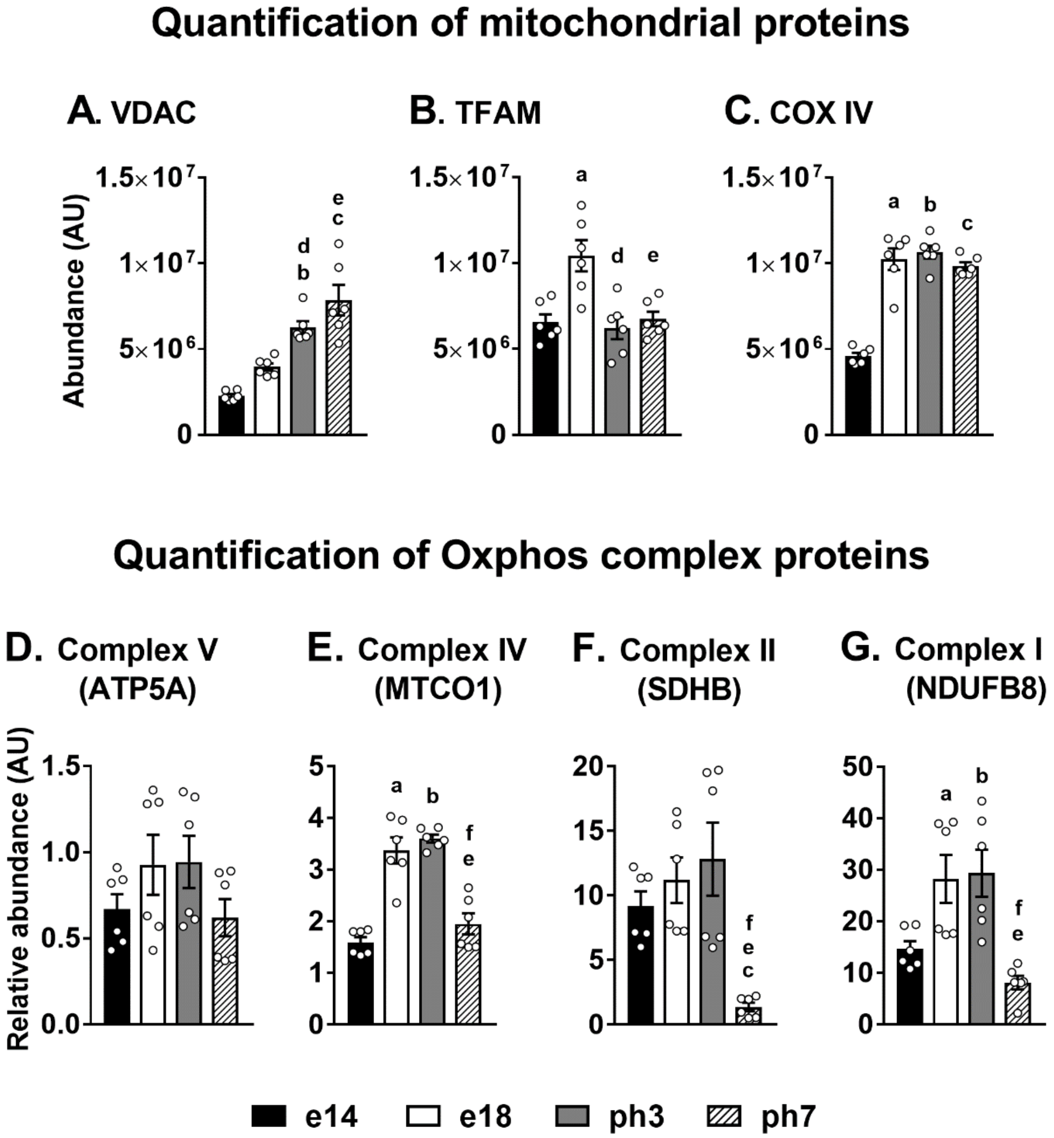

Supplementary Fig S3. Graphical representation of mitochondrial proteins and Oxphos complex proteins. Densitometry of mitochondrial proteins (**A**) VDAC (**B**) TFAM (**C**) COX IV (from Fig 4B).and (**D-G**) the Oxphos complex proteins (from Fig 4G). n = 6/ group; Means were considered significant at P ≤ 0.05 following pairwise mean comparison, represented by the following alphabets. 'a'- e14 vs. e18; 'b' - e14 vs. ph3; 'c' - e14 vs. ph7; 'd' - e18 vs. ph3; 'e' - e18 vs. ph7; 'f' - ph3 vs. ph7.

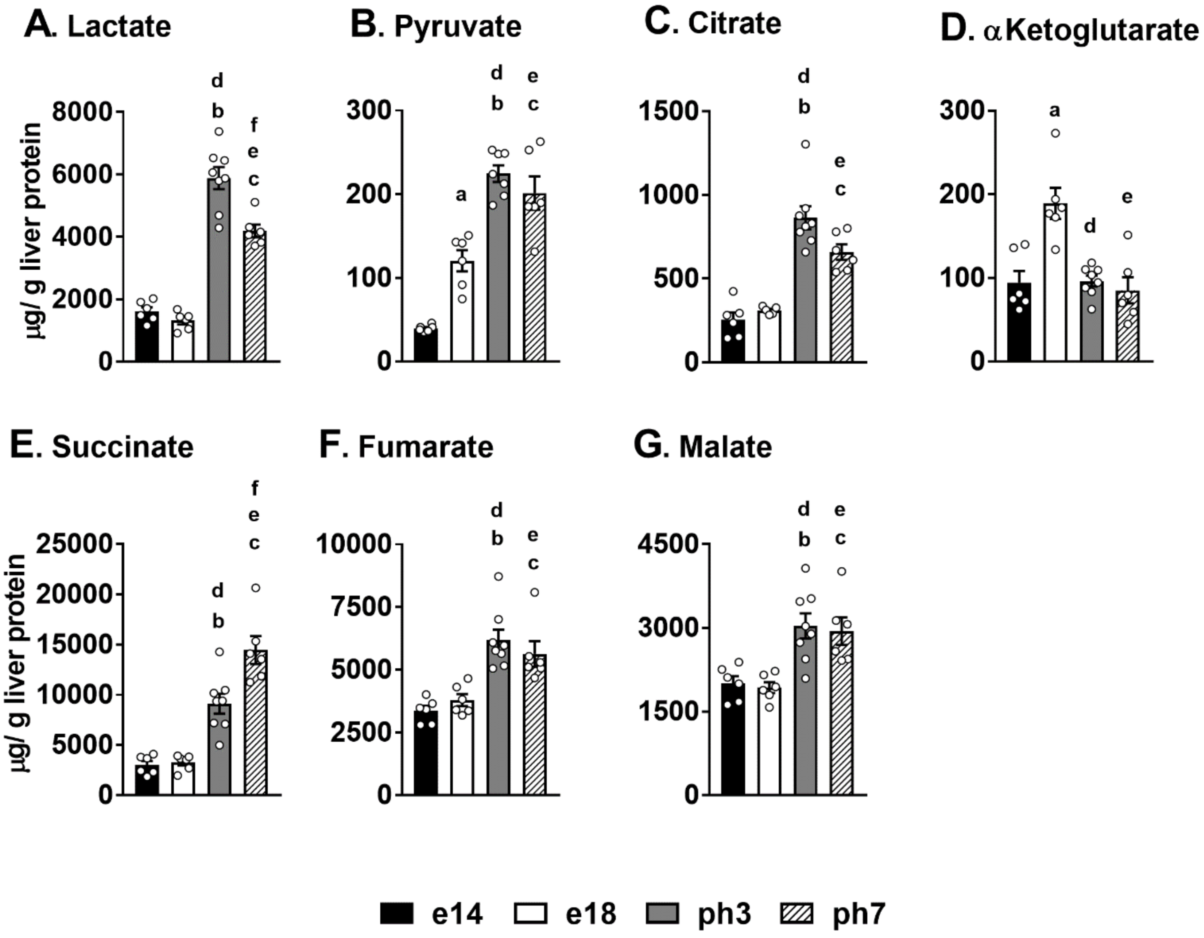

Supplementary Fig S4. Concentrations TCA cycle intermediates in the liver.

Changes in concentrations of (**A**) Lactate (**B**) Pyruvate (**C**) Citrate (**D**) Lactate (**E**) Succinate (**F**) Fumarate (**G**) Malate. n = 6/ group; results were considered significant at P ≤ 0.05 following pairwise mean comparisons, represented by the following alphabets. ‘a’- e14 vs. e18; ‘b’ - e14 vs. ph3; ‘c’ - e14 vs. ph7; ‘d’ - e18 vs. ph3; ‘e’ - e18 vs. ph7; ‘f’ - ph3 vs. ph7.
